# Alternative Treatments for Zirconium Oxide to Compare Commonly Used Surface Treatments to Determine Which Has the Least Effect on the Phase Transformation

**DOI:** 10.3390/ma17215175

**Published:** 2024-10-24

**Authors:** Beata Śmielak, Leszek Klimek

**Affiliations:** 1 Department of Prosthodontics, Medical University of Lodz, ul. Pomorska 251, 92-231 Lodz, Poland; 2 Institute of Materials Science and Engineering, Lodz University of Technology, 90-924 Lodz, Poland; leszek.klimek@p.lodz.pl

**Keywords:** zirconium dioxide, surface treatments, phase transformation

## Abstract

Traditional mechanical processing of zirconium leads to an unfavorable transformation, from a metastable tetragonal phase to a monoclinic phase (t→m), which weakens the structure of the material and subsequently leads to damage to the prosthetic restoration. The aim of this research is to compare commonly used surface treatments to determine which has the least effect on t→m. Thirty cylindrical samples made of sintered zirconium were divided into six groups based on the following treatments: polishing, grinding, sandblasting, chemical etching, laser structuring or dry plasma etching. After surface treatment, the samples were subjected to the following tests: X-Ray Diffraction, microscopic examination, surface wettability and surface roughness measurements. Chemical etching, laser structuring and plasma etching significantly reduce the content of the monoclinic phase. All surface treatments significantly reduced the final amount of the monoclinic phase. However, chemical etching did not provide sufficient surface roughness. Both laser and plasma processing offer the advantage of creating structural patterns on the surface of elements. However, as plasma etching requires a mask to obtain the appropriate pattern on the surface, it seems that laser processing offers more and varied structuring possibilities. Laser structuring is easier to control and more economical than the other methods.

## 1. Introduction

Zirconium dioxide is a polymorphic ceramic material without the addition of glass. Zirconium oxide crystals form fine grains of 0.2–0.5 μm [1] and exist as three allotropic varieties: monoclinic (m), tetragonal (t) and cubic (c). At room temperature, zirconium oxide occurs in the monoclinic variety. When heated to a temperature above 1170 °C, it changes into the tetragonal version, and above 2370 °C, into the cubic version [2,3]. Polymorphic transformations are diffusion-free, athermal, occur at the speed of sound, involve volume changes and depend on the grain size [4]. The most advantageous from the point of view of mechanics is the tetragonal form, which remains stable at high temperatures. In the 1970s, methods were developed to maintain the tetragonal variety at room temperature by adding oxides of magnesium, calcium, yttrium or cerium [5].

Zirconium dioxide is highly refractory (melting point 2700 °C), relatively resistant to corrosion in an acidic environment, has high hardness (12.17–13.7 GPa) and mechanical strength, good insulating properties and a low ability to absorb ionizing radiation. It also has a very low porosity coefficient, i.e., it has a very uniform microstructure, practically without empty spaces or micro-gaps, indicating that it is a non-absorbent material. Moreover, it shows high wear resistance and low thermal conductivity [6,7,8]. Its bending strength ranges from 840 to 1200 MPa [1,9], i.e., the highest value of all materials used to produce all-ceramic tooth restorations [10]. Its stress intensity coefficient (K1C) is 9–10 K_IC_ MPa^1/2^, this being the amount of energy needed to initiate fracture of the material sample (so-called brittle fracture), reflecting its resistance to crack propagation [10]. It has a Young’s modulus, i.e., elasticity or stiffness, of 210–224 GPa [1,10,11]. It is also a relatively light material compared to noble metals such as gold (19 g/cm^3^) or platinum (21 g/cm^3^), with the density being 5.56 g/cm^3^ for the monoclinic variety and 6.1 g/cm^3^ for the tetragonal variety [10,11].

The mechanical properties of 3Y-TZP depend largely on the grain size [3,4,12,13,14,15,16,17]. Smaller grain sizes (<1 µm) are more stable and less likely to be susceptible to spontaneous temperature changes; however, above a critical grain size, this stability decreases [18]. However, transformations do not occur at grain sizes below a certain limit (≈0.2 µm), leading to a reduction in bending strength [19]. The final grain size depends on the sintering processes [2], with higher sintering temperatures leading to larger grain sizes [13,14,17]. Also, grain size and the subsequent phase stability of the 3Y-TZP are also influenced by significant temperature differences [5]. In the tetragonal phase, the grains remain in a metastable state. Each propagating crack in the structure slightly stretches the material and causes the local transformation of unstable tetragonal grains into monoclinic ones, resulting in an increase in grain volume of up to 3–5%; this process closes the resulting gap in the ceramic near the crack tip. This also limits the possibility of the propagation of microcracks in the structure of zirconium dioxide, thus strengthening the material [20]. In turn, spontaneous transformations of the tetragonal into the monoclinic phase may also lead to the formation of internal stresses, microcracks and, consequently, to the weakening of the structure and damage to the veneering porcelain [21,22]. Studies show many cases of veneering porcelain chipping (15–62%), cracks (25–50%), delaminations (>10.7%) and large fractures (3–33%) [23,24,25].

The zirconium oxide ceramic framework is also difficult to process. This is because the zirconium oxide-based ceramic surface is non-reactive, hard and resistant to most conventional processing methods. To improve the bond strength between the zirconium oxide framework and veneering ceramics, attempts are made to develop the surface by mechanical processing with various types of rotary tools, abrasive blasting, etching with strong acids and, rarely, laser surface structuring and plasma etching [26]. Due to its high hardness, the material requires very aggressive action. In turn, aggressive processing promotes the formation of microcracks, which lead to unfavorable transformations [27,28,29]. In the case of zirconium oxide-based ceramics, the issue of abrasive blasting is controversial. Some authors recommend it because it increases the strength of the connection with the veneering ceramics [27,30]. Others question it because it weakens the bond with ceramics due to unfavorable transformations, LTD and microcracks in the material [29,30]. Others suggest that it is not needed at all [31]. In turn, chemical etching using 5% or 9.5% hydrofluoric acid (HF) gives unsatisfactory results and allows for obtaining minimal roughness on the nanometer scale [32].

Some publications report that zirconium oxide is a suitable material for laser processing due to its low thermal conductivity. Arami et al. [33] examined the influence of three different types of lasers (Er:YAG, Nd:YAG and CO_2_) and Al_2_O_3_ sandblasting on the surface roughness parameters Ra, Rku and Rsk. Er:YAG laser treatment yielded similar Ra roughness parameters to sandblasting. In turn, Ersu et al. [34] compared CO_2_ laser treatment and sandblasting, with different abrasive gradations and pressure values, on Ra; the highest Ra was noted after laser processing (2.41 μm). Nikolas et al. [35] investigated the effect of XeCl (wavelength 308 nm), KrF (wavelength 248 nm) and ArF (wavelength 193 nm) gas lasers with a 15–20 ns pulsed mode on the surface modification of zirconium oxide. A sharp change in roughness occurred when the energy of the pulse entering the amplifier per unit area, i.e., the fluence, of the laser exceeded 0.7 J/cm^2^ at a wavelength of 248 nm at the beginning of melting. However, despite many attempts, no clear recommendations exist regarding the use of laser processing to obtain appropriate structuring for zirconium oxide suitable for binding with veneering ceramics. It is known that both the type of cavities and their shape depend on the selection of appropriate laser parameters [36,37].

The effect of plasma etching on zirconium oxide processing has not been fully explored. In a plasma, charged particles interact with external electric and magnetic fields and with each other; this leads to the formation of its own electric field, with the plasma becoming a conductive plasma [38,39]. Plasma technology can be used to clean contaminated surfaces, or to etch or cover surfaces with protective coatings, e.g., hydrophobic ones. The efficiency of the process is largely influenced by the appropriate selection of the process gas, for example, for surface cleaning [38,40]. Plasma can be used for physical or chemical etching. The physical type involves high-energy ions knocking out atoms of the substrate material, while the chemical one involves the creation of volatile compounds. Chemical etching of a material in a plasma occurs when the interaction between its particles and the surface of the material leads to the formation of a stable volatile compound; the most commonly used gasses are SF6, CHF3, CF4 and C4F8, which are sources of fluorine [40]. Physical plasma etching acts by bombarding the target, e.g., ZrO_2_, with ions coming from the plasma. As a result, the O-Zr-O bonds are broken. Oxygen from the layer is released into the atmosphere, and the broken bond combines with ions or particles present in the plasma, forming O-Zr-F bonds [40,41]. During plasma etching, various types of masks with different aperture sizes can be placed on the surface of the etched material to create a structure on the surface. These can be made of stainless steel, aluminum, copper or nickel: the latter do not react with fluorine radicals originating from plasma at the temperatures used for etching processes.

One of the basic features of a surface that can be assessed is its physical parameters [42,43]. One important feature is roughness, which at the micro level is a factor that can improve the quality of the connection of materials. Appropriately produced rough surfaces can support the distribution of stresses on the surface, increasing energy dissipation during the action of forces breaking the connection of materials [42]. In dental prosthetics, the development of the foundation surface of crowns and bridges plays a key role in ensuring a good connection with the veneering material [11,43,44,45,46,47,48,49]. Modifications of various types of prosthetic elements are based on the analysis of the geometric structures present in them and on the size of the surface contact angles and the associated surface free energy. It is very important to examine the thickness of the transformation layer after processing. As the transformation phase spreads from the sample surface through its entire volume, microcracks and residual stresses may develop and lead to a decrease in bending force [48,49,50,51].

The aim of this research is to compare commonly used surface treatments to determine which has the least effect on the phase transformation.

## 2. Materials and Methods

Thirty cylindrical samples made of zirconium oxide 3Y-TZP (Ceramill Zi; Amann Girrbach AG, Koblach, Austria) were cut by milling; the content of individual oxides is shown in Table 1. After cutting, the surfaces of the sample bases were ground on a grinder–polisher (Struers S.A.S.; Champigny sur Marne, France) to create a uniform surface. The grinding was performed successively with sandpaper with grits of 120, 240, 360, 500, 800, 1200 and 2500, and then polished with a 3 µm diamond suspension. The prepared samples were sintered in a furnace (Ceramill Therm; Amann Girrbach AG, Koblach, Austria) using a universal program (8°/min from 200° to 1450°, two hours at a constant temperature of 1450° and appropriate cooling time). The sintering process took about 10 h. The material shrinkage was approximately 21%. After sintering, the samples had the following dimensions: diameter 25 mm, height 8 mm.

After the sintering process, the samples were divided into five groups of five samples each. The surfaces of the cylinder bases in each group were subjected to one of the following treatments:

- Polishing using a polishing wheel with a 3 μm diamond suspension.
- Grinding (with water cooling) with abrasive disc diamond grit no. 120 (106–125 µm).
- Abrasive blasting (Mikroblast Duo; Prodento-Optimed, Warsaw, Poland) with Al_2_O_3_ grains (size 110 µm). For the Mikroblast Duo device, the following parameters were used: pressure *p* = 0.4 MPa; distance from sanding nozzle tip to sample surface 10 mm; stream angle 45 degrees; processing time 20 s. After processing, the samples were cleaned with compressed steam, washed in deionized water in an ultrasonic bath for eight minutes and dried with compressed air.
- Chemical etching in 40% hydrofluoric acid for 30 min (experimentally prepared in laboratory).
- Laser structuring using a Nd:YAG laser (Fidelis; Fotona, Ljubljana, Slovenia); wavelength 1070 nm; average power 15 W; pulse duration 100 ns; pulse frequency 25–125 kHz. Structuring consisted of cutting grooves 30 µm wide at 100 µm distances.
- Dry plasma etching in SF6 gas for 60 min: substrate polarization voltage 600 V; pressure 4.5 Pa; SF6 gas flow rate 10 sccm. During the etching process, a stainless steel mesh mask (AISI 304) was placed on the surface (mesh size 35 μm).

The samples were then subjected to the following tests:

### 2.1. X-Ray Diffraction (XRD)

X-ray Diffraction (XRD) was performed using an Empyrean X-ray diffractometer (Malvern Panalytical Ltd.; Malvern, UK) to determine the phase composition. The tests were conducted using a copper lamp (CuKα: 1.54056 Å) with the X-ray tube power supply parameters U = 40 kV and I = 45 mA. The number of tetragonal and monoclinic phases was calculated based on the Garvie and Nicholson (G-N) method [4].

### 2.2. Microscopic Examination

Differences in surface topography were observed using a scanning electron microscope (SEM S-3000N; Hitachi High Technologies Corp, Tokyo, Japan). The acquisition of microscopic images was performed in the “light” of secondary electrons.

### 2.3. Surface Wettability

Surface free energy (γ_s_) was determined by measuring the contact angle using the Krüss GmbH, Hamburg, Germany model FM40 EasyDrop device and Drop Shape Analyzer software: Drop Shape Analysis (https://www.kruss-scientific.com/en/know-how/glossary/drop-shape-analysis, accessed on 22 September 2024). Two measuring liquids were used: distilled water and diiodomethane. The liquids were dispensed in 0.3 μL amounts. The individual dispersion (γd_s_) and polar (γp_s_) components of the tested samples (*n* = 5) were calculated based on the Owens–Wendt model [51]. The calculated components were used to determine the mean surface free energy.

### 2.4. Surface Roughness Measurements

Surface roughness measurements were performed using a Mitutoyo SJ-410 (Mitutoyo Polska Sp. z o.o.; Wrocław, Poland) apparatus in accordance with the ISO 21920-3:2021 standard [52].

## 3. Results

### 3.1. The XRD Test Results Are Presented in Figure 1a–f and in Table 2

The presented diffractograms show that reflection from the monoclinic phase appears in samples after individual surface treatments, indicating a partial transformation from the tetragonal phase. The phases were identified based on the PDF 4 database (tetragonal phase—card no. 00-042-1164; monoclinic phase—card no. 00-036-0420). The diffractograms show changes in the intensity of reflections in the processed samples, which is due to the different content of individual phases in the tested samples. The obtained diffractograms were used to determine the content of the tetragonal and monoclinic phases in samples after individual surface treatments. The results are presented in Table 2.

**Figure 1:**
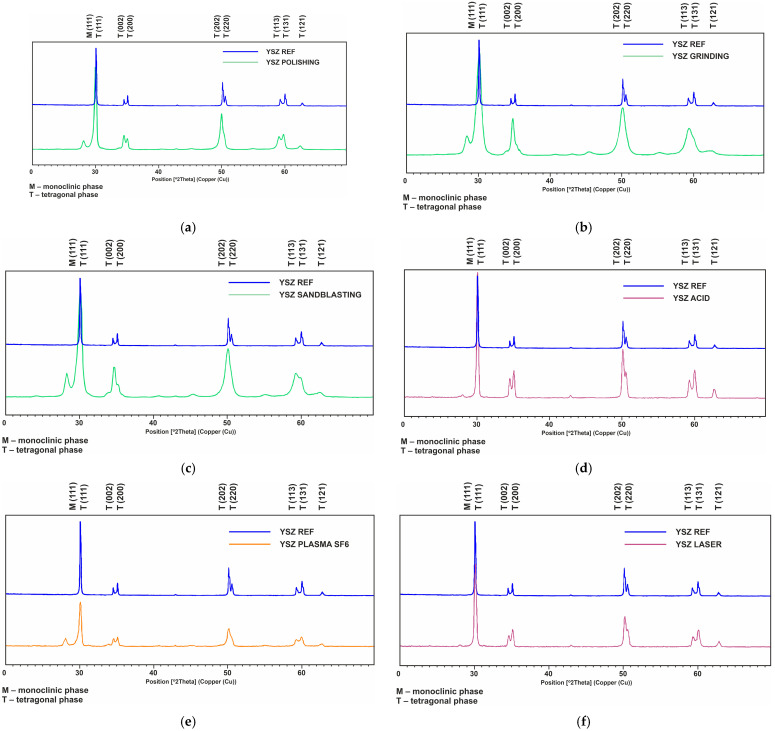
Diffractogram of the sample surfaces: (**a**) after polishing, (**b**) after grinding, (**c**) after abrasive blasting, (**d**) after etching, (**e**) after laser structuring, (**f**) after plasma etching.

**Table 2:** The share of the tetragonal and monoclinic phases according to processing variant, as identified in diffraction studies; values calculated using the G-N method.

Treatment	Phase Content [% mass]	
	Tetragonal	Monoclinic
Polishing	83.2	16.8
Grinding	83.1	16.9
Sandblasting	78.2	20.8
Chemical etching	98.2	1.8
Laser structuring	92.1	7.9
Plasma etching	95.2	4.8

The largest share of the monoclinic phase ranged from 20.8 to 16.8 (% by weight), and was observed after polishing, grinding and sandblasting. The lowest phase transformation, 0.8 to 7.9 (% by weight), was associated with chemical etching, followed by plasma etching and laser structuring.

### 3.2. The Microscope Images of the Surface of Zirconium Oxide Samples after the Various Sample Processing Methods (500× and 2000× Magnification) Are Shown in Figure 2a–f

As indicated in the images, significant differences can be seen between the samples following individual treatments. As expected, the polished surface is very smooth; its geometric surface structure and that of the abrasive blasting sample do not show directionality. In contrast, the ground surface is directional, with the scratches arranged in one direction according to the grinding direction. Directional structures were noted for the laser-structuring surface, running in the direction the grooves were cut, and the plasma-etched sample, but in two perpendicular directions.

**Figure 2:**
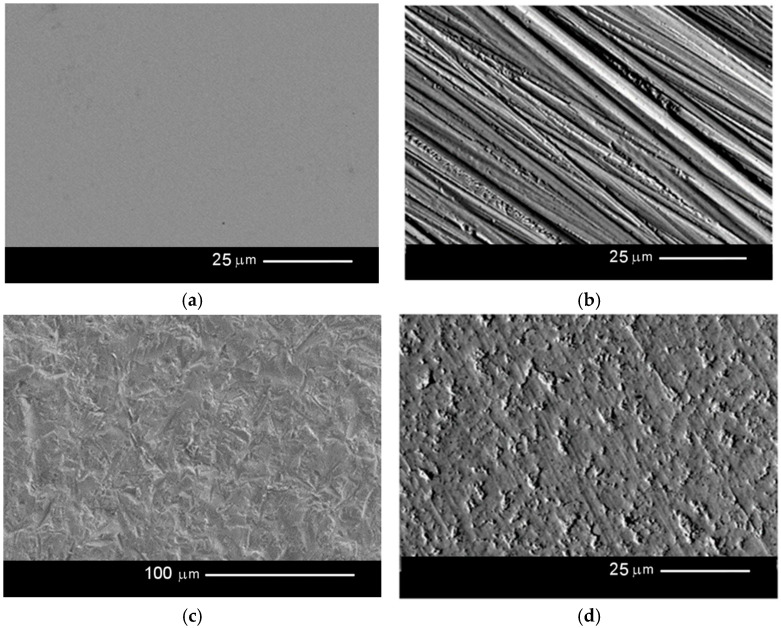

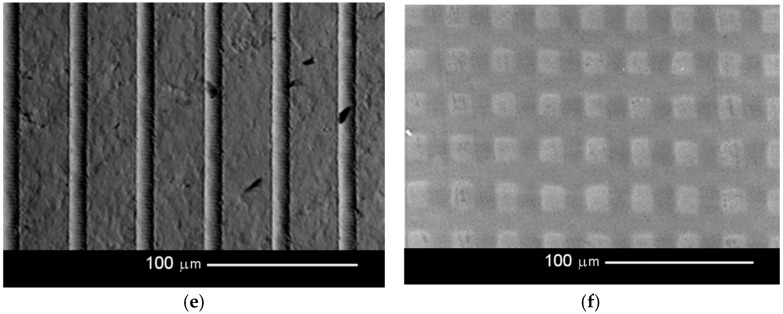
Microscope images of the sample surfaces: (**a**) after polishing, magnification 2000×; (**b**) after grinding, magnification 2000×; (**c**) after abrasive blasting, magnification 500×; (**d**) after etching, magnification 2000×; (**e**) after laser structuring, magnification 500×; (**f**) after plasma etching, magnification 500×.

### 3.3. The Results of Measurements of Surface Wettability with Water and Diiodomethane after Various Variants of Sample Processing Are Shown in Figure 3(1a–2f); Contact Angle Measurement Results Are Shown in Table 3; Wettability Results: Free Energy (SEP) Measurements Are Shown in Table 4

It can be seen that the treatments had differing influences on surface wettability. As for the polar liquid (water), the best wettability (30.3 ± 1.6 deg) was achieved by abrasive blasting, and the worst (101.5 ± 0.7 deg) by chemical etching.

Although all surfaces are wettable with an apolar liquid (contact angle < 90 deg), the best wettability was achieved after chemical etching (47.4 ± 0.6 deg). Similar contact angles were achieved by laser structuring and plasma etching. The lowest wettability was obtained by abrasive blasting (68.0 ± 0.8 deg).

**Figure 3:**
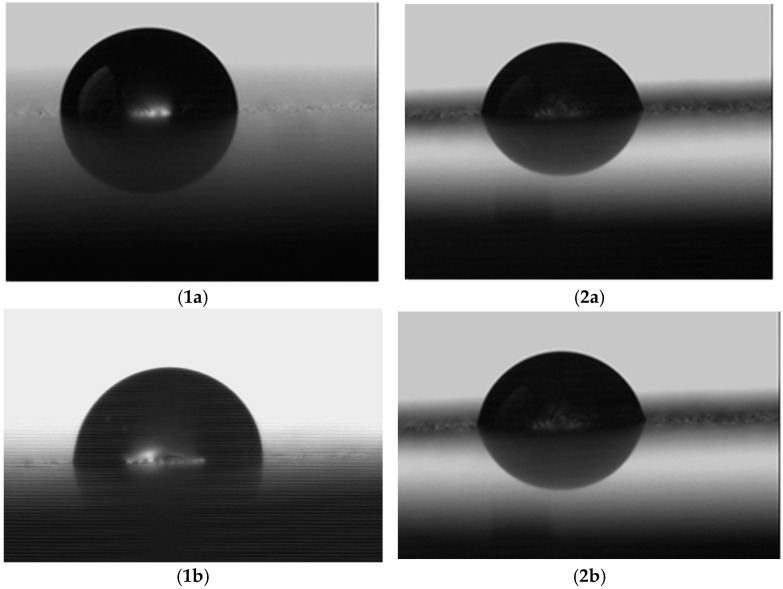

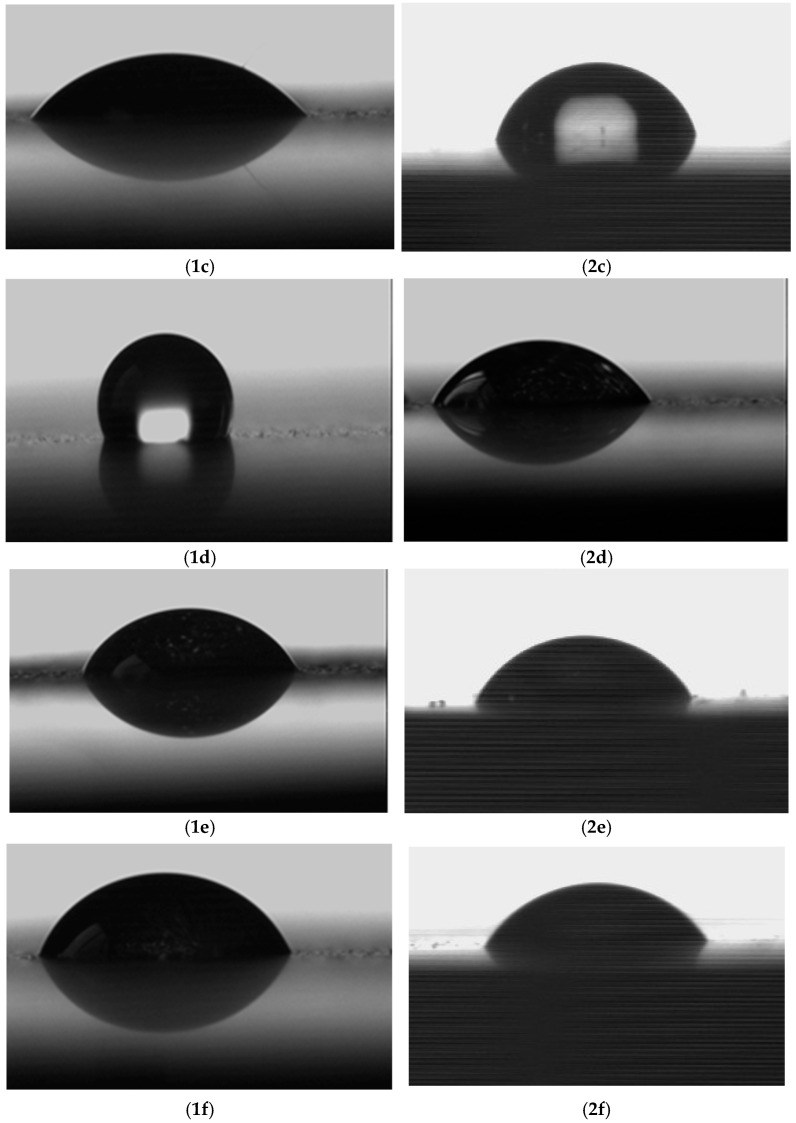
Wetting of the surface of samples by a drop of (**1**) water and (**2**) diiodomethane after (**a**) polishing, (**b**) grinding, (**c**) abrasive blasting, (**d**) etching, (**e**) laser structuring, (**f**) plasma etching (will be added after correction).

**Table 3:** Results of contact angle measurements.

Sample	Q_w_ [deg]	Q_j_ [deg]
polishing	86.8 ± 2.3	57.5 ± 2.2
grinding	69.8 ± 1.0	54.1 ± 0.5
sandblasting	30.3 ± 1.6	68.0 ± 0.8
chemical etching	101.5 ± 0.7	47.4 ± 0.6
laser structuring	50.3 ± 1.5	51.1 ± 1.4
plasma etching	52.2 ± 1.6	49.2 ± 1.1

**Table 4:** Free energy (SEP) measurements.

Sample	Component		SEP [mJ/m^2^]
	Polar [mJ/m^2^]	Dispersive [mJ/m^2^]	
polishing	4.0 ± 0.3	26.7 ± 2.2	30.7 ± 2.3
grinding	13.5 ± 1.8	24.8 ± 0.4	38.4 ± 1.7
sandblasting	52.6 ± 2.8	11.8 ± 0.2	64.4 ± 3.0
chemical etching	0.9 ± 0.2	37.1 ± 0.1	38.0 ± 0.3
laser structuring	27.2 ± 3.4	23.1 ± 0.4	50.3 ± 3.8
plasma etching	24.9 ± 0.6	24.4 ± 0.1	49.3 ± 0.7

The surface free energy also varies depending on the type of surface treatment. The highest value was observed after abrasive blasting (64.4 ± 3.0), and the lowest after polishing (30.7 ± 2.3).

### 3.4. The Surface Roughness Measurements Are Shown with Regard to Sample Processing Method in Figure 4a–f

The presented surface profiles show the differences in the depth of the obtained surface structures. The polished and chemically etched samples show surface irregularities of the order of 0.1 µm; the depressions and elevations of the structure elements above the baseline are within 5 µm. In both cases, the sizes of the depressions and hills have similar values.

**Figure 4:**
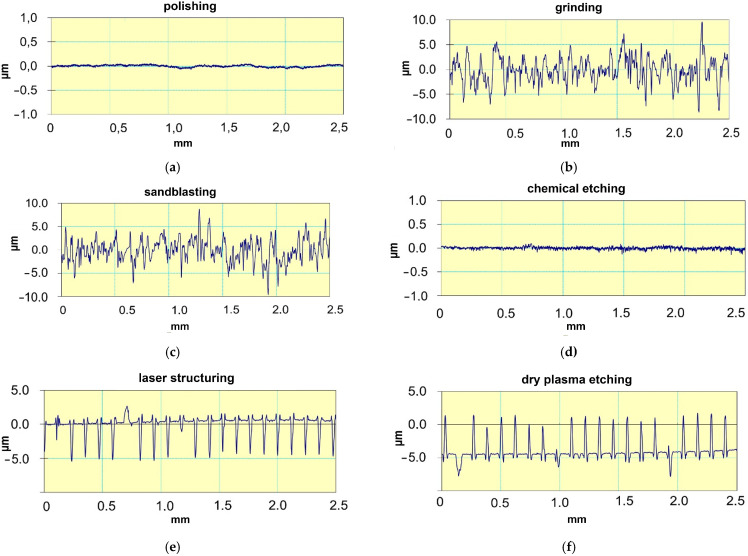
Roughness profiles: (**a**) after polishing, (**b**) after grinding, (**c**) after sandblasting, (**d**) after etching, (**e**) after laser structuring, (**f**) after plasma etching.

## 4. Discussion

The long-term bond strength between zirconium and veneering ceramic may be influenced by the unfavorable transformation of the tetragonal phase into the monoclinic phase [29,31,44,52,53,54]. Our findings indicate that the highest share of the monoclinic phase (20.8% by weight) was recorded after sandblasting, with slightly lower levels observed after grinding (16.9%) and after polishing (16.8%). This is most likely related to the mechanical induction of the transformation. When considering abrasive blasting, attention should also be paid to the processing time, which was not addressed in the conducted research: the amount of monoclinic phase has been found to increase with processing time [55].

It should be noted that an unfavorable phase transformation also occurred after abrasive processing by grinding and polishing. However, in some cases it is difficult to give up grinding, as it may be necessary to fit the prosthetic to the limits of the preparation, with an appropriate shape, and fit the occlusal conditions in the oral cavity. It is not always possible to predict and solve all these issues during computer design. However, polishing may not always be necessary: it certainly does not contribute to improving the connection and may only have esthetic value.

By far, the smallest phase transformation was recorded for etching, laser structuring and dry plasma etching, where the amount of the monoclinic phase was from 7.9 to 0.8 [% by weight]. Based on the degree of phase transformation, the most advantageous treatment would be chemical etching, which demonstrated the least amount of monoclinic phase conversion (1.8%), followed by plasma etching (4.8%) and laser structuring (7.9%). Much higher transformations were noted for the other treatments, ranging as high as 20%.

The microscopic examinations carried out showed that grinding created scratches on the surface of the samples, which were partially smoothed by polishing. The surface treated by abrasive blasting and HF acid etching became irregular with non-directional surface irregularities, while laser and plasma treatment created ordered and directional structures.

Wettability and surface free energy are important parameters that may indicate the quality of the subsequent connection of the prosthetic framework with the veneering ceramics. Liquids with high wettability flow well in surface irregularities. In the case of liquid veneering ceramics, good wettability allows for an increase in the mechanical composition of the joint.

The best wettability with a polar liquid is demonstrated by the surface after abrasive blasting (contact angle = 30.3 ± 1.6 deg), followed by those treated with laser structuring (50.3 ± 1.5 deg) and plasma etching (52.2 ± 1.6 deg). Higher contact angles (lower wettability) were noted for surfaces after other treatments, but the polished surface is at the wettability limit (86.8 ± 2.3 deg), and the chemically etched surface should be considered non-wettable (101.5 ± 0.7 deg), i.e., contact angle > 90 deg. Hence, the ceramic suspension in water, which will be applied to the zirconium oxide framework, will flow best into the surface irregularities created by abrasive blasting, and followed by those formed by laser structuring and chemical etching. In the case of an apolar liquid (diiodomethane), the best wetted surface is that treated by chemical etching (contact angle 47.4 ± 0.6 deg). Slightly higher values were found for other treatments and were more or less similar: plasma etching −49.2 ± 1.1; laser structuring, 51.1 ± 1.4. Although abrasive blasting appears to have a significantly greater impact (68.0 ± 0.8 deg) on bond quality, the activity of the liquid ceramic at the firing temperature will have a strong influence, as the apolar liquid will flow into the surface irregularities. As such, chemical etching and then plasma etching and laser structuring seem to be the most beneficial. The obtained wettability results were obtained on model liquids, which show the behavior of polar and dispersive liquids on the tested surfaces, and these are necessary to determine the surface free energy. In practice, it is important to know how the liquids behave in conditions other than those tested, for example, in the case of veneering ceramics, how they behave at elevated temperatures (firing temperatures). To obtain a more complete picture, the research should be extended to include changes in wettability with temperature [56]. Surface free energy can be treated as a measure of the reactivity of the surface; the greater the reactivity, the greater the component of the chemical connection. The highest surface free energy was recorded by the sample after abrasive blasting (64.4 ± 3.0 mJ/m^2^), followed by those after laser structuring (50.3 ± 3.8 mJ/m^2^) and plasma etching (49.3 ± 0.7 mJ/m^2^). Surfaces after other treatments had lower surface free energy values, ranging from 30 to 38 mJ/m^2^. To ensure a strong connection between the framework and the veneering ceramics, the most suitable processing method is abrasive blasting, followed by laser structuring and plasma etching. In addition to the final surface free energy value, the values of individual components are also important. Similarly to the process of wetting the surface with polar and apolar liquids, the value of the polar component may be important when applying ceramics, which are applied as mixtures with water: a polar liquid. In addition, the dispersion component value should be important when firing ceramics, i.e., when the water is removed. The liquid ceramics formed during firing are an apolar liquid. The values of these components are related to the contact angles of individual liquids. Therefore, in the case of the polar component, the highest values were recorded for the surface after abrasive blasting (52.6 ± 2.8 mJ/m^2^), followed by laser structuring (27.2 ± 3.4 mJ/m^2^), plasma etching (24.9 ± 0.6 mJ/m^2^) and grinding (13.5 ± 1.8 mJ/m^2^). The lowest values were obtained after chemical etching (0.9 ± 0.1 mJ/m^2^) and polishing (4.0 ± 0.3 mJ/m^2^). The values of the dispersive components were not as diverse as those of the polar components. The highest value was achieved by the sample surface after chemical etching (37.1 ± 0.1 mJ/m^2^), and the lowest after abrasive blasting (11.8 ± 0.2 mJ/m^2^). The other treatments yielded similar dispersion components, i.e., 20–30 mJ/m^2^. Taking into account their values for surface free energy and its components, such treatments as polishing, grinding and chemical etching should be excluded for preparing the surfaces of the zirconium oxide framework for veneering ceramics. Abrasive blasting works the best, followed by laser structuring and chemical etching, which have similar values.

The geometric structure of the treated surfaces depends on the treatment used. To obtain a proper connection between the veneering ceramics and the prosthetic framework, it is necessary for the liquid veneering ceramics to flow into the surface irregularities. To make this possible, these irregularities need to be of appropriate dimensions: if they are too narrow, inflow may be prevented by capillary effects, and if they are too shallow, anchoring may be weakened. Previous works found abrasive blasting to obtain the most favorable roughness parameters for joint strength, more precisely, an abrasive grain with a gradation of 110 µm and pressure of 0.4 MPa [23,57]. Therefore, it should be assumed that the surface roughness after this treatment is optimal. The heights and depths of the irregularities created by this treatment are of the order of 5 µm. Similar unevenness was obtained by grinding. This was to be expected because the abrasive material used for blasting and the coating on the grinding disc used similar grain sizes. Although the laser-cut grooves also have the same depth, their width is greater than that of the irregularities after abrasive blasting. However, this is not a problem, because it is possible to adjust the depth and width of the grooves and the spacing between them during laser treatment. Smaller irregularities were observed for the other treatments. In the case of plasma etching, the etched areas have depths of the order of 1 µm, which is about five times smaller than in previous treatments. However, this does not seem to be a problem, because deeper etching can be achieved by changing the plasma parameters and extending the time. The only problem in obtaining the appropriate spacing and width of etchings may be the availability of a masking mesh. Polishing and chemical etching achieved irregularities of the order of tenths of a micrometer. Such shallow irregularities cannot ensure sufficient mechanical anchoring of the ceramics in the prosthetic framework. Theoretically, the acid digestion time could be extended. However, taking into account the etching time used in this work (30 min), in order to obtain surface roughness analogous to that in abrasive blasting, the process would have to take at least several hours, and would not be cost-effective.

Among the tested surface treatments, the one that induced the least degree of transformation from the tetragonal to monoclinic phase was found to be chemical etching, followed by plasma etching and laser structuring. Phase contents of a few percent can be considered acceptable. However, chemical etching does not achieve appropriate roughness, and hence unsatisfactory wettability and surface free energy values. Other treatments achieved much higher levels of transformation, with some reaching 20%. This may be unfavorable in the long term, resulting in the appearance of cracks in the framework, weakening the connection with the veneering ceramics, and thus increasing chipping [12,30,45,51,52,53,54]. Overall, the best methods for structuring the surface of the zirconium oxide framework appear to be the lasers and the plasma etching treatments. They also offer the advantage that the arrangement and orientation of the obtained structures can be adjusted to accommodate on the expected loads.

## 5. Conclusions

1. Of the tested treatments, 40% HF etching, laser structuring and plasma etching significantly reduce the content of the monoclinic phase in the treated zirconium oxide surface.
2. Although abrasive blasting ensures proper connection of the veneering ceramics with the framework, it also results in considerable transformation of the tetragonal phase into the monoclinic phase, which may cause damage to prosthetic elements in the long term.
3. Laser structuring and plasma etching allow some degree of control over the geometries of surface structures (dimensions, orientation), thus allowing them to be adapted to the direction of the expected load.
4. Recommended surface treatments are laser structuring and plasma etching.
5. Laser structuring is easier to control and more economical than the other methods.

## Figures and Tables

**Table 1:** Chemical composition 3Y-TZP (Ceramill Zi).

Chemical Composition (Ceramill Zi)	
Mass Percentage	
ZrO_2_ + HfO_2_ + Y_2_O_3_	≥99
Y_2_O_3_	4.5–5.6
HfO_2_	≤5
Al_2_O_3_	≤0.5
Other oxides	≤1

## Data Availability

The original contributions presented in the study are included in the article, further inquiries can be directed to the corresponding author.
